# Correction to “Random control selection for conducting high‐throughput adverse drug events screening using large‐scale longitudinal health data”

**DOI:** 10.1002/psp4.13198

**Published:** 2024-06-27

Chiang, C. W., Zhang, P., Donneyong, M., Chen, Y., Su, Y., & Li, L. (2021). Random control selection for conducting high‐throughput adverse drug events screening using large‐scale longitudinal health data. *CPT: Pharmacometrics & Systems Pharmacology* 10(9):1032‐1042. https://doi.org/10.1002/psp4.12673

In the published version of this article, the co‐first author's name Pengyue Zhang was misspelled as Penyue Zhang.

The published article has also been corrected to reflect this change.

We apologize for this error.

